# Giving Back: A mixed methods study of the contributions of US-Based Nigerian physicians to home country health systems

**DOI:** 10.1186/s12992-016-0165-9

**Published:** 2016-06-14

**Authors:** Joseph Nwadiuko, Keyonie James, Galen E. Switzer, Jamie Stern

**Affiliations:** Johns Hopkins School of Medicine, 4640 Eastern Avenue, Baltimore, MD 21202 USA; Graduate School of Public Health, University of Pittsburgh, 130 De Soto Street, Pittsburgh, PA 15213 USA; Department of Medicine, Psychiatry, and Clinical and Translational Science, University of Pittsburgh, 3501 Forbes Ave, Pittsburgh, PA 15213 USA; Department of Medicine, University of Pittsburgh, 3459 Fifth Avenue, Pittsburgh, PA 15213 USA

**Keywords:** Brain drain, Diaspora, Health workforce, Globalization, Migration

## Abstract

**Background:**

There is increased interest in the capacity of US immigrants to contribute to their homelands via entrepreneurship and philanthropy. However, there has been little research examining how immigrant physicians may support health systems and what factors facilitate or raise barriers to increased support.

**Methods:**

This study used an observational design with paper questionnaire and interview components. Our sample was drawn from attendees of a 2011 conference for US Based Nigerian physicians; respondents who were not US residents, physicians, and of Nigerian birth or parentage were excluded from further analysis. Respondents were randomly selected to complete a follow-up interview with separate scripts for those having made past financial contributions or medical service trips to support Nigerian healthcare (Group A) and those who had done neither (Group B). Survey results were analyzed using Fischer exact tests and interviews were coded in pairs using thematic content analysis.

**Results:**

Seventy-five of 156 (48 %) individuals who attended the conference met inclusion criteria and completed the survey, and 13 follow-up interviews were completed. In surveys, 65 % percent of respondents indicated a donation to an agency providing healthcare in Nigeria the previous year, 57 % indicated having gone on medical service trips in the prior 10 years and 45 % indicated it was “very likely” or “likely” that they would return to Nigeria to practice medicine. In interviews, respondents tended to favor gifts in kind and financial gifts as modes of contribution, with medical education facilities as the most popular target. Personal connections, often forged in medical school, tended to facilitate contributions. Individuals desiring to return permanently focused on their potential impact and worried about health system under-preparedness; those not desiring permanent return centered on how safety, financial security and health systems issues presented barriers.

**Conclusions:**

This study demonstrates several mechanisms by which health systems may benefit from expatriate engagement. Greater identification of reliable local partners for diaspora, deeper collaboration with those partners and a focus on sustainable interventions might improve the quantity and impact of contributions. Ethnic medical associations have a unique role in organizing and facilitating diaspora response. Public-private partnerships may help diaspora negotiate the challenges of repatriation.

## Background

There is a significant gap in resourcing for health systems in many low and middle income countries (LMICs). In 2009 a World Bank/UNICEF/UNFPA report estimated that $68.9 billion dollars needed to be invested in LMIC health systems in order to ensure the achievement of the Millennium Development Goals (MDGs) [1]. In terms of human resources, an estimated gap of 2.4 million physicians, nurses, and midwives has prevented achievement of the MDGs, 84 % of which is accounted by Africa and Southeast Asia [2].

As of 2010, there were an estimated 265,851 physicians in the United States (US) who have received their education in other nations, 128,729 of whom came from low income countries [3]. Home country health systems have suffered from the gap left by emigrated health professionals. This was most crystallized right before the recent Ebola epidemic in Liberia, where more than 50 % of nationally trained physicians were practicing overseas as of 2004 [4], and the nation had only 51 physicians in 2014 to care for 5 million individuals [5]. Other studies have demonstrated an increase in HIV mortality related to physician emigration [6].

Conversely, home country health systems may potentially benefit from the involvement of “diaspora” (i.e. emigrated) health professionals. Unfortunately very little research has attempted to understand the level, impact, or factors related with diaspora contributions towards home country healthcare. Case studies have attempted to measure the work of individuals or collective single contributions such as humanitarian trips [7–9]. One study has examined the philanthropy of Zimbabwean physicians but was focused largely on remittances and family directed gifts in kind [10]. (Research into philanthropy by nonmedical diaspora, on the other hand, is by no means new. Although the large majority of literature focuses on the economic impact of remittances on development, there has been growing recognition of the capacity of diaspora to contribute and volunteer directly to social and humanitarian causes [7, 11–14]).

The literature on permanent return by health workers is slightly more expansive, but still inconclusive. Existing studies on Peruvian and Pacific Islander returnees identify remigration related “pull” factors of family and potential impact in their home country, while lost potential earnings and bureaucracy at home raise barriers to return and retention [15–17]. India is believed to have experienced increased return migration of health workers (due in part to improved economic opportunities), and a number of encouraging studies have investigated the return of nurses to their homeland [18]. On the other hand, some studies suggest that permanent return is unlikely for the majority of physicians [19, 20]. One study of South African physician émigrés in Canada echoed a resounding disinclination towards return—or any type of post-emigration engagement [21].

Given the significant needs of LMIC health systems and the recorded willingness of diaspora to contribute generally, there is need for greater research to examine the involvement of diaspora health workers in origin country health systems. With 3271 physicians, Nigerians constituted the largest component of sub-Saharan Africa-trained physicians in the United States in 2011 (equaling 44 % of all sub-Saharan Africa trained physicians in the US) [22]. The following study takes a mixed methods approach composed of a questionnaire and follow-up interviews to determine the capacity in which a group of US-based Nigerian physicians support Nigerian health, including (but not limited to) financial contributions, medical service trips, and permanent return. Our principal hypotheses were that US-Based Nigerian physicians with strong relationships with Nigerian medical institutions (exhibited by correspondence frequency) and a strong belief in their effectiveness as practitioners would be more likely to contribute via donations and medical service trips while physicians with family members in Nigeria would exhibit a stronger desire to relocate to Nigeria to practice medicine.

## Methods

### Design

This study used an observational mixed methods research design utilizing a questionnaire and semi-structured interviews. A convenience sample of US-practicing physicians of Nigerian birth or heritage practicing in the United States were recruited at the 2011 annual convention of the Association of Nigerian Physicians in the Americas (ANPA), the largest association of Nigerian physicians practicing in the U.S. Permission to recruit was obtained from ANPA. All attendees could submit the questionnaire but only respondents who were both US residents and physicians were considered for analysis. Participants were asked to complete a questionnaire and indicate on the form if they were willing to receive follow-up correspondence to complete an in-depth interview. Physicians who completed the questionnaire were eligible to be placed in a raffle for one of two Apple iPads.

Of those who agreed and requested follow-up, interviewees were randomly selected to from two subgroups: those having made a financial contribution in the previous year or a medical service trip in the past 10 years to Nigeria (“Group A”), and those having done neither (“Group B”). Participants from both groups were contacted via phone or email to schedule a 30 min follow-up interview, which was conducted over the phone by the principal investigator. Interview participants were not compensated for their involvement. The interview guide consisted of two scripts, one each for Group A and Group B participants. Both scripts asked follow-up questions about participants’ desire (or lack thereof) to re-immigrate to Nigeria, as determined by the initial questionnaire. Interviews were audiotaped and then transcribed verbatim by study investigators.

### Instruments

A 31-item questionnaire was designed to examine opinions and behaviors related to Nigerian healthcare as well as demographic and professional characteristics. The questionnaire was created in conjunction with a psychometrician (G.S.) and reviewed for clarity and relevance of items by five US-Based African physicians. For interviews, two semi-structured scripts were written: “Group A” scripts inquired into the type of contribution, discovery of contribution opportunities, rationale for contributing, and perceived effect of contribution. “Group B” scripts inquired into reasons for not contributing as well as what interviewees felt would influence them to contribute more. Both scripts included a subset of items asking interviewees why they desired to re-immigrate to Nigeria (or not) and, if applicable, what they felt needed to change before they actually would re-immigrate. Interview scripts were piloted for clarity and relevance by three US-based African physicians.

### Analysis

Questionnaire results were analyzed using Fischer tests. Outcome variables included whether monetary donations were provided to agencies providing healthcare in Nigeria in the previous year; whether medical service trips were made to Nigeria in the previous 10 years; the level of desire to return to Nigeria for medical practice, and the perceived likelihood of permanent return for medical practice.

Interview data were analyzed using thematic content analysis. Thematic content analysis is used in qualitative research to facilitate comparative analysis of respondents’ accounts from interview transcripts and notes in order to identify themes that are common in the data [23]. The principal investigator initially read each transcript and designated codes based on recurring themes and a second investigator read the transcripts and reviewed the initial codes for discrepancies. The two investigators then coded each interview using the finalized codebook, cross-checking each coded interview for relevancy and consistency. Survey results were analyzed with STATA and interviews and codes were analyzed by Atlas.ti 7. All components of the study was reviewed and approved by the University of Pittsburgh Institutional Review Board.

## Results (Table 1)

**Table 1:** Demographics

Sex	
Female	29 (39 %)
Age	
Mean (Standard Deviation)	51 (8.9)
Marital Status	
Single/never married	3 (4 %)
Married/marital like	62 (83 %)
Divorced/Widowed	6 (8 %)
Not entered	2 (3 %)
Dependents	
# w/Children under 18 or other dependents at home	52 (70 %)
Born In Nigeria	66 (88 %)
Relatives in Nigeria	69 (92 %)
Studied Medicine in Nigeria	60 (80 %)
Years in US	
< 10 years	5 (7 %)
11–20 years	26 (35 %)
21–30 years	33 (44 %)
31–40 years	8 (11 %)
> 40 years	3 (4 %)

### Survey results

Seventy-five of 156 (48 %) individuals who attended the conference met inclusion criteria and completed the survey. Sixty-five percent of respondents reported having made a donation to an agency providing healthcare to Nigeria in the previous year, and 29 % of respondents reported having given more than $1000. Fifty-seven percent of respondents reported going on a medical service trip to Nigeria in the previous 10 years, with 23 % of respondents reporting having gone on four or more.

The likelihood of making a medical service trip was associated with the frequency of correspondence with medical institutions within Nigeria (*p* = .01), the perceived effectiveness of Nigerian medical agencies (*p* = .039), their perception of their medical training in Nigeria (*p* = .033), and their perception of physical security in Nigeria (*p* = .005). Physicians’ frequency of meeting other Nigerian professionals was associated both with their likelihood of having made a donation (*p* = .024) or a medical service trip (*p* = .001). No personal characteristics (including sex, age, marital status, income, presence of relatives in Nigeria, time spent in US) were significantly associated with any outcomes.

#### Remigration to Nigeria

Fifty-five percent of respondents expressed at least a moderate desire to re-emigrate to Nigeria to practice medicine, and 45 % indicated it was “very likely” or “likely” that they would do so. Individuals who more clearly perceived of a method of contribution to employ after returning to Nigeria reported a greater desire to return (*p* < .001). Higher belief in the impact of a respondent’s practice in Nigeria was associated with increased desire (*p* < .001) and perceived likelihood (*p* = .002) of return. The perception of economic security while practicing medicine in Nigeria was also associated with increased likelihood of return (*p* = .03). Again, no personal factors were related to personal desire or likelihood of migration to Nigeria.

### Interviews (Tables 2 and 3)

**Table 2:** Engagement measures

Donations (in previous year)	No Donations	$1–250	$251–500	$501–1000	$1001–2000	>$2001
	26 (35 %)	8 (11 %)	9 (12 %)	10 (13 %)	10 (13 %)	12 (16 %)
Medical Service Trips (in previous 10 years)	0	1	2	3	>4	
	31 (41 %)	6 (8 %)	14 (19 %)	6 (8 %)	17 (23 %)	
Desire of Return	Not at all	A little bit	Moderately	Very Strongly		
	15 (20 %)	18 (24 %)	15 (20 %)	26 (35 %)		
Likelihood of Return	Very Unlikely	Unlikely	Likely	Very Likely		
	19 (25 %)	22 (29 %)	15 (20 %)	19 (25 %)

**Table 3:** Questionnaire significant associations

	Any Donation in the previous Year	Any Medical Service Trip in the previous 10 years	Stated desire to relocate to Nigeria	Stated likelihood of relocating to Nigeria
Impression of training in Nigeria	0.046	0.033	--	
Frequency of encounters with Nigerian professionals	0.024	0.001	--	--
Frequency of correspondence with Nigerian medical institutions	--	0.010	--	--
Amount of time spent in Nigeria (out of previous 2 years)	0.025	0.002	--	0.020
Number of medical service trips to Nigeria in previous 10 years	0.029		--	---
Perceived likelihood of medical trips in next 2 years	--	0.009	<0.001	0.002
Perception of method of contribution to Nigeria	--	--	<0.001	
Perceived effectiveness of Nigerian medical agencies		0.039		
Perception of impact of practice in Nigeria	--	--	<0.001	0.002
Perception of presence (or absence) of sufficient physical security for medical practice in Nigeria	--	0.005	--	----
Perception of presence (or absence) of economic security for medical practice in Nigeria	--	--		0.031
Stated desire to relocate to Nigeria	--	--		<0.001
Stated likelihood of relocating to Nigeria	0.008	---	<0.001	

Values computed using Fisher’s exact test. Non-significant values excluded

#### Type and Recipients of Contributions

Interviews identified a significant portion of physicians who contributed via financial as well as non- financial, non-clinical means, particularly via gifts-in-kind, medical education and patient- or system-level consultations. For the large majority of interviewees, contributions were directed towards medical education facilities, which received diaspora-conducted lectures, journals, diagnostic equipment, and scholarship funding. Other recipients included other healthcare organizations, clinician colleagues in Nigeria, other diaspora physicians going on medical service trips, and at times, ANPA itself. Interviewees’ stated reasons for contribution stemmed directly from physicians’ identification of needs in Nigeria, as well as a sense of nationalism and self-identification with the institution to which their contribution was directed. In some cases, their realization of Nigerian health systems needs actually grew as a result of their own integration into the US health system, as they began to contrast their resources in the US with those of colleagues in Nigeria.

Interview respondents indicated that opportunities to contribute, more often than not, were sourced from physicians’ social and professional networks in Nigeria or the United States. The inverse also held true, as individuals who did not contribute often attributed it to a lack of personal connections to facilitate contributions. This particularly was the case for individuals who did not attend medical school within Nigeria and felt they lacked the history needed to identify needs and complete contributions there. Individuals also tended to trust their networks (including alumni networks in particular) to facilitate contributions rather than the government or at times even local Nigerians.

#### Challenges to contributions

In interviews, respondents frequently cited health systems level problems as barriers to contributions, including a perceived lack of capacity in the health sector (particularly for subspecialty driven interventions), mismanagement concerns, bureaucratic obstacles, or a perceived lack of interest by local officials in diaspora contributions. Government was also identified as a barrier for many of the same reasons, although several respondents still recognized that government was an essential partner for achieving the large scale health goals desired by the diaspora.

#### Perceived effect of contributions

When asked, many physicians could name examples where recipients benefited from their contributions. In one case, a pooled alumni gift provided a server and multiple computers to a university; another respondent emphasized the impact of his annual pediatric lecture series at his alma mater. Nonetheless several respondents also expressed reservations that their contributions were adequate to deal with Nigeria’s public health crises. Many instead felt that their efforts would only be effective with the support of larger-scale partners such as the government.

#### Group B respondents

Of the six respondents in Group B--identified as not having given financially in year prior to the questionnaire or made medical service trips in the prior 10 years--three had indeed given financially (although it is not known whether it was in the prior year or not) and one had made a donation between the initial questionnaire and the interview. Others had been involved in lower scale contributions such as medical consultations or academic support with individual colleagues in Nigeria.

When asked about barriers to contributions, Group B respondents identified many of the same governmental and health system barriers mentioned by respondents in Group A. The majority of Group B respondents had trouble conceptualizing a useful way to contribute or lacked connections with institutions or individuals deemed reliable to facilitate contributions. A number mentioned projects they had envisioned to improve treatment in their field of practice but could not complete because due to lack of interest from their counterparts within Nigeria. Finally, a lack of time and perceptions of insecurity in Nigeria were also cited as contribution barriers.

#### Return

Of the 13 interviewed, three indicated a desire to return to Nigeria with plans to either engage in administrative work, in a consultative capacity, and in medical education. One respondent was in the process of building a diagnostic center in Nigeria he planned to run full time. When asked of the potential benefits of relocation, respondents focused on the potential impact of their practice in Nigeria but worried that system-wide dysfunction or lack of government support would throttle their effectiveness. Those same health system concerns were shared by those not wishing to migrate, although safety and family concerns were also indicated by the latter group.

## Discussion

The transnationalism approach to modern migration holds, amongst other things, that technological advancements have recently added a new dimension to migrants’ dual identity in new and former countries, permitting them to invest more heavily in homelands “abroad” as well as here in the US [24]. This dynamic plays itself most clearly in remittances, which totaled $21billion in Nigeria in 2014 and can account for as much as 20 % of GDP in small nations like Liberia [25]. This study demonstrates that these “investments” can likewise be captured directly by the health sector. Emigrated physicians might already benefit from their familiarity with language, cultural practices and health systems realities in their countries of origin, particularly if they trained in those countries. The pressing question for these diaspora revolves around which appropriate strategies they should employ as they engage and how their contributions can improve in quantity and quality.

### Individual level engagement

It is not insignificant that 16 % of this sample gave more than $2000 the prior year towards Nigerian healthcare, particularly when considering the country’s GDP per capita in 2011 (the year the survey was collected) to be $2507. Of note, a mail survey conducted by Clemens revealed that US and Canadian-based African-trained physicians remitted more than $6500 on average annually [26]. While the proportion of these remittances actually directed to health systems is unknown, those results are consistent with the relatively high levels of donations in this study.

It is been estimated that the emigration of Nigerian trained physicians equates to a loss of $89,238 per physician to Nigeria in terms of subsidized education costs [27]. Various studies have suggested that these funds either be recouped via physician-levied taxes [28] or by intergovernmental payments from receiving to sending countries [29]. This study demonstrates some evidence that diaspora physicians’ contributions to health systems might represent returns on sending countries’ educational investment, although such contributions may not always be directly made to medical education institutions or even financial in nature. Of note, a literal repayment of this amount would be equivalent to installments totaling $2230 a year over an assumed 40 productive years. This study unfortunately shows only a fraction of physicians making this level of financial donations to health systems in a single year. However, this figure still provides a useful (and attainable) benchmark for diaspora contribution levels.

There are various pathways to increase health-system directed donations. First, diaspora networks themselves have an important role in exposing members and nonmembers to reliable contribution opportunities (as discussed further below). International nongovernmental organizations (NGOs) may also help “broker” contributions to small local NGOs that they identify as being effective in order to counter fears of financial mismanagement, which came up frequently in this study. AfricaResponds is an example of such an initiative, employing this strategy to support organizations tackling the Ebola response in West Africa [30]; international donor agencies may also match these donated funds for added impact. Government issued “diaspora bonds” are a final option that have been advocated to fund health delivery, but trust would need to be reestablished between the members of this sample and public institutions in order for this to be successful [31].

Other non-pecuniary contributions such as gifts-in-kind, medical service, and transfer-of-knowledge activities were also popular within this sample. It is likely that the high prevalence of gifts in kind in this sample is itself an adaptation to perceived lack of financial accountability within health systems. However, gifts in kind as well as medical service trips carry a number of risks of their own that should be properly identified and navigated in the context of long-term partnership. Several guidelines incorporating these goals have been published elsewhere [32–37]. In fact, education-driven trips and contributions, such as those conducted by respondents in this study for their medical alma maters, might prove to be cheaper to execute with more sustained effects [34]. The value of contributions through all these modalities could be potentially measured by the cost of delivery to the expatriate physician, or more appropriately, according to disability-adjusted life-years averted by interventions [32].

### Network-level engagement

Both quantitative and qualitative components of this study demonstrated a clear link between engagement in compatriot networks—formal and informal--and physician engagement, suggesting that these networks might have a role to play in fostering contributions. This holds significance in light of the proliferation of “ethnic medical groups”, of which there are at least 66 in the US according to a list compiled by the American Medical Association [38]. These groups have the potential to guide individual contributions and facilitate group-wide responses. As mentioned above, if the exposure and support provided by networks is indeed an important predeterminant of contributions, it might be reasonable to believe that more intensive outreach from networks might increase homeland contributions by diaspora both within and outside their membership. (By extension the same may be said about alumni groups from international medical schools, as well as hometown associations to support initiatives in more rural locations [39].)

The collective leverage lent by a network approach can also be used to partner with home country governments to build health systems capacity, as in 2011 when the Nigerian Ministry of Health affirmed a memorandum of understanding with ANPA as well as the Nigerian Universities Commission which allows, among other items, their participation in the overhaul of the medical education curriculum within Nigeria [40, 41].

A final application of the network approach for diaspora stems from recent perspectives emphasizing the importance of politics and political leadership in achieving healthcare advancement and particularly universal health coverage [42, 43]. Diaspora, particularly those who have maintained citizenship in their countries of origin, are at a unique advantage to advocate for policies that promote health care equity and quality via channels created by their respective networks.

International NGOs can participate at any point in this continuum as partners to ethnic medical associations for the purpose of increasing their capacity to intervene in any of these arenas. Several collaborations have been made between ethnic organizations and NGOs such as Oxfam-Novib, the Ford Foundation, and the National Alliance of Latin American and Caribbean Communities [11].

### Permanent relocation

The fact that a significant proportion of respondents desired to return and practice medicine in Nigeria is worthy of attention. While this might be attributed to the selection of the sample itself, it should be noted that other surveys have recorded return desire to be as high as 70 % amongst African physicians [44]. The mean age of those in our sample indicating a moderate-to-high likelihood of return (51, standard dev. 8) should also be noted, as these professionals carry a premium of experience, professional contacts, and potential productive years.

Of course there are many barriers between imagined and actual return, including financial sustainability, security, supply chain deficits, and bureaucratic obstacles. By adopting a private (or even non-profit) approach, diaspora might be able to innovate to adapt to non-security related challenges and provide efficient care outside of governmental mechanisms. One study demonstrated that the proportion of physicians working the private sector was inversely related to rates of expatriation within a series of countries [45]. Public-private partnerships in LMICs however remain irreplaceable as a means to maintain private for-profit venture survival as well as equitable access for patients, as has been shown in other studies [46].

## Limitations

This study is limited by sample size, a potential selection bias and the fact that interview respondents represent a spectrum of contributory levels instead of clear “contributor” and “non-contributor” categories. More detail regarding the personal contributions of diaspora network members, as well as physician migrants not involved in diaspora groups, should be investigated. However, we feel that this study sample represents an important component of diaspora upon whom interventions are likely to have the greatest impact, and that this study represents an important initial step towards categorizing the modes and channels of contributions, as well as identifying interventions to increase those contributions.

## Conclusions

There is an important component of diaspora engaged in home country health care utilizing a wide spectrum of contributory levels and mechanisms, with a preferred target of medical education institutions. Ethnic medical groups have a role in increasing these contributions and prove useful collaborators in achieving large-scale health goals. Outside organizations can partner with diaspora to advise individual and network-level contributions. For diaspora preparing to permanently relocate to their countries of origin, the private sector might offer an attractive option, although governmental support remains necessary for their successful integration.

## Abbreviations

ANPA, Association of Nigerian Physicians in the Americas; LMICs, Low and Middle Income Countries; NGO, Non-Governmental Organization; UNFPA, United Nations Population Fund; UNICEF, United Nations Children’s Fund; USA, United States of America
